# A Comprehensive Description of the Roadmap to Identify and Validate a Myelin Biomarker

**DOI:** 10.1177/11772719251349605

**Published:** 2025-07-06

**Authors:** Giovanna Capodivento, Davide Visigalli, Andrea Armirotti, Chiara Demichelis, Marinella Carpo, Roberto Fancellu, Erika Schirinzi, Daniele Severi, Diego Franciotta, Fiore Manganelli, Gabriele Siciliano, Alessandro Beronio, Elisabetta Capello, Paola Lanteri, Eduardo Nobile-Orazio, Angelo Schenone, Luana Benedetti, Lucilla Nobbio

**Affiliations:** 1 UO Clinica Neurologica, IRCCS Ospedale Policlinico San Martino, Genoa, Italy; 2 DINOGMI, University of Genova, Genoa, Italy; 3 Analytical Chemistry Lab, Fondazione Istituto Italiano di Tecnologia, Genoa, Italy; 4 UOC Neurologia, ASST Bergamo Ovest, Treviglio, Italy; 5 UO Neurologia, IRCCS Ospedale Policlinico San Martino, Genoa, Italy; 6 Department of Clinical and Experimental Medicine, Neurological Clinic, University of Pisa, Italy; 7 Department of Neurosciences, Reproductive, and Odontostomatological Sciences, University Federico II, Naples, Italy; 8 UO Laboratorio di Patologia Clinica, APSS Provincia Autonoma di Trento, Italy; 9 Department of Neurology, Sant’Andrea Hospital, La Spezia, Italy; 10 Neurophysiology Center, IRCCS Istituto Neurologico Carlo Besta Foundation, Milan, Italy; 11 Neuromuscular and Neuroimmunology Service, IRCCS Humanitas Clinical and Research Institute, Department of Medical Biotechnology and Translational Medicine, Milan University, Italy

**Keywords:** biomarker, myelin, sphingomyelin, CIDP, GBS

## Abstract

**Background:**

Demyelination and remyelination are major issues for scientists dealing with myelin disorders in both clinical and research fields. Despite that, rapid, reliable and convenient tools to monitor myelin changes still lack both in central and peripheral nervous system. Given that myelin is enriched in specific lipids and proteins, it is reasonable they could represent eligible candidates as structural damage biomarkers for this characteristic membrane. Among them, we focused on sphingomyelin (SM) due to the enrichment in myelin and because it is easily measurable in different biological matrices.

**Objective:**

Depicting the roadmap to identify and validate SM dosage as a myelin biomarker useful for pre-clinical and clinical practice.

**Design:**

This study adheres to STROBE guidelines for observational cross-sectional studies on human patients and to ARRIVE guidelines for animal models.

**Method:**

Following the recommendations of the Society for CSF Analysis and Clinical Neurochemistry, we describe the stepwise process to validate SM as a myelin biomarker, starting from the optimization of the fluorescence-based assay and analytical validation in experimental models until clinical and pathological validation in biological fluids of neurological patients.

**Results:**

SM dosage monitors myelination, demyelination, remyelination and even small myelin changes associated to myelin pathology and pharmacological treatments in experimental models. SM is detectable in human biological fluids and informative of myelin damage in the CSF of neurological patients. SM dosage identifies myelin breakdown in the CSF of patients affected by Guillain-Barrè Syndrome (GBS) and Chronic Inflammatory Demyelinating Polyradiculoneuropathy (CIDP), identifying disease activity, axonal from demyelinating variants, and avoiding misdiagnosis.

**Conclusion:**

SM dosage displayed extremely promising real-word performances being able to identify, monitor and stage myelin pathology. Given that it is simple, inexpensive and easily adaptable to routine use in any hospital setting, it might rapidly progress to the implementation and impact on clinical outcomes.

## Introduction

The myelin sheath is a greatly extended and modified lipid-enriched plasma membrane that is wrapped around the axon in a spiral fashion in both peripheral nervous system (PNS) and central nervous system (CNS). Myelin is composed of a specific set of lipids that accounts for at least 70% of its dry weight, and it is increasingly evident that monitoring myelin lipidome is informative of nervous system integrity or injury.^1-3^ To this end, diseases in which myelin is originally affected, damaged, or destroyed represent conditions that can be realistically identified and monitored by a structural lipid biomarker.

Among possible candidates of this last, we focused on complex sphingolipids because they are highly abundant in myelin membrane and their metabolism is finely tuned and regulated during myelin maturation.^3-7^ Qualitative and quantitative analysis of sphingolipids in biological samples is usually performed by mass spectrometry combined with liquid chromatography. These methods are very sensitive but also quite expensive and require specialized expertise making them unsuitable for daily clinical practice.^8^ Indeed, the technology and translation gap is one of the major problems in biomarker development process.^9,10^ For this reason, among sphingolipid species we selected sphingomyelin (SM) which couples a specific enrichment in myelin with the ease of quantification by a fluorescence-based assay.^1,5-7,11,12^

Here, we describe the actions performed to identify and validate SM dosage as a myelin biomarker, following the key phases recommended by the Society for CSF Analysis and Clinical Neurochemistry.^9,10^

The first steps of our pipeline provides for technical and analytical validation in different well-known experimental models of myelin pathology. In particular, we demonstrated that SM monitors dysmyelination in the CMT1A rat and demyelination in EAE mice, as a grounded proof-of-concept for SM dosage as a marker of myelin changes in PNS and CNS myelinopathy.^13-16^

After proving our hypothesis that SM is able to capture myelin status in ex vivo and in vitro models, we moved to human biological fluids of neurological patients.

To this end, we detailed the steps of technical and analytical validation, including agreement analysis with high-resolution mass spectrometry. The linear relationship of SM levels quantified by our assay with those measured using this technique is worthy of emphasizing. Given that SM assay is inexpensive and suitable to routine clinical use, it overcomes the technical translational gap which represents an upper limit in the process of biomarker development.^10^

Due to this very promising analytical performance, we finally moved to the clinical validation, hypothesizing and demonstrating that demyelination at tissue level might be detected by SM dosage in the CSF of patients affected by immune-mediated demyelinating neuropathies, namely Guillain-Barrè Syndrome (GBS) and Chronic Inflammatory Demyelinating Polyradiculoneuropathy (CIDP). According to the recommendations of CSF society, a mandatory requirement before clinical implementation is a strong validation in wide and independent cohorts of patients.^10^ To achieve this goal, here SM data from neurological patients from different neurological centers, enrolled for retrospective and prospective studies, were merged and reanalyzed.

In this large cohort of patients our assay unambiguously identifies demyelination typical of active CIDP and Acute Inflammatory Demyelinating Polyneuropathy (AIDP). SM levels differentiate active from stable stage of CIDP, fundamental to select the most appropriate timing for therapeutic intervention. Moreover, SM dosage avoids misdiagnosis of CIDP with 100% specificity which still represents a major issue for the correct identification of CIDP patients.

Overall, this study allowed to demonstrate that SM assay encompasses most of the ideal characteristics of a biomarker representing a promising tool to the real-word monitoring of myelin changes in a wide spectrum of conditions.

## Materials and Methods

### Experimental Models

#### Animal Model of Peripheral Dysmyelination

Transgenic Sprague-Dawley rats affected by an inherited peripheral dysmyelinating disease (ie, Charcot-Marie-Tooth type 1A, CMT1A OMIM:118220, n = 8) and wild-type (WT, n = 8) rats were used for the experiments.^14,17,18^ Following euthanasia with carbon dioxide (CO_2_), sciatic nerves and brain were dissected from the rats and processed for biochemical and neuropathological studies. We confirm that all methods were performed in accordance with relevant guidelines/regulations. In particular, the research protocols presented in this study are conducted in accordance with the ARRIVE guidelines and are included in those reviewed and approved by the IRCCS Ospedale Policlinico San Martino OPBA (Institutional Animal Welfare Body) and by the Italian Ministry of Health (project number approval: 798/2016-PR; Supplemental File 1).^19^

#### Animal Model of Central Demyelination

Experimental autoimmune encephalomyelitis (EAE) was induced in female C57BL/6 mice (n = 9; aged 6-8 weeks) by the administration of MOG (peptide 35-55) emulsion of Freund’s complete adjuvant, together with pertussis toxin.^20^ A matched control group (CTRL, n = 4), injected with the same emulsion of Freund’s complete adjuvant together with pertussis toxin but without MOG was used. We followed daily, for 15 days after immunization with MOG or control emulsion, clinic and weight of both groups. Moreover, clinical disease was scored from 0 to 5 with 0 being healthy, 1 complete loss of tail tonicity, 2 loss of righting reflex, 3 partial paralysis of a limb, 4 complete paralyzes of 1 or both hind limbs, and 5 moribund. Clinical signs of disease are usually first observed between days 10 and 15, followed by the chronic phase of disease, characterized by severe walking disability and progressive gray matter atrophy. We sacrificed mice by CO_2_ euthanasia at the acute inflammatory phase of the disease (around 15 days post-immunization). CTRL mice at the same time points were also sacrificed. Spinal cord and sciatic nerve were dissected from these animals and immediately frozen in liquid nitrogen to quantify sphingomyelin (SM). We confirm that all methods were performed in accordance with the ARRIVE guidelines and are included in those reviewed and approved by the IRCCS Ospedale Policlinico San Martino OPBA (Institutional Animal Welfare Body) and by the Italian Ministry of Health (project number approval 949/2016-PR).^19^

#### In Vitro Model of Peripheral Demyelination/Remyelination

A demyelination/remyelination experimental setting established in myelinating dorsal root ganglia (DRG) cultures was used.^21^ DRG cultures from 15-day-old wild-type rat embryos were prepared as previously described.^22^ In particular, DRG cultures were established to include the following experimental conditions: (i) induction of demyelination by addition of Forskolin (Fsk, 20 μM (n = 6) and 40 μM (n = 6; Sigma-Aldrich, F6886) for 10 days; (ii) control cultures treated with vehicle (ie, dimethyl sulfoxide, DMSO, Sigma-Aldrich, D8418, n = 6) for 10 days; (iii) induction of remyelination by withdrawal of Fsk 40 μM following 10 days to induce remyelination until the end of the experiment, (n = 6). DRG cultures were then carefully rinsed with Dulbecco’s Phosphate Buffer Saline (DPBS, Invitrogen, 14190-144), and used for immunofluorescence staining of the myelin sheath and SM dosage (see below). To quantify SM, cultures were scraped and recovered from the culture dishes by centrifugation. Resulting pellets were handheld homogenized in 75 μL of 0.25% Triton X-100 (Sigma-Aldrich, T8787) and stored at −80°C until lipid extraction and SM dosage.

#### Neuropathology

##### Quantitative Ultrastructural Analysis in Myelinating Dorsal Root Ganglia (DRG) Cultures

Organotypic DRG cultures were fixed and processed for myelin basic protein (MBP) immunofluorescence staining as previously described.^14^ Morphometric evaluation of the digitized images was performed using an ad-hoc Image Pro-Plus macro we established in our laboratory, using the Image Pro-Plus Software (Immagini e Computer, Rho, Milan, Italy). To quantify MBP, we calculated the percentage of myelinated area expressed as the area of pixels positive for the red staining of MBP compared to the total area of the acquired image.

##### Quantitative Ultrastructural Analysis in Rat Sciatic Nerves

Light microscopy morphometric analysis was performed on the contralateral sciatic nerves of those used to quantify SM from the same CMT1A and WT rats. Sciatic nerves were dissected and immediately fixed in 2.5% glutaraldehyde (Sigma-Aldrich, G7776) in cacodylate buffer (0.15 M), pH 7.4 (Sigma-Aldrich, C4945), for 24 hours. Samples were embedded in Epoxy resins (Sigma-Aldrich, 45359-1EA-F) and prepared for semithin sections, which were stained with toluidine blue. Morphometric analysis was performed in WT (n = 8) and CMT1A (n = 8) rats from 3 different littermates on at least 800 myelinated fibers/animal. To cover almost completely each nerve fascicle, 25 frames were collected at x1000 magnification from each sample, digitalized, and stored using the Image Pro-Plus Software (Immagini e Computer, Rho, Milan, Italy). Data from fibers with evident degeneration signs or that have been sectioned in a longitudinal way were not collected. We established an ad-hoc automated macro to evaluate different myelin parameters including the amount of myelinated area.^14,23^

### Human Patients

In this study, we reanalyzed and merged data from different cohorts of patients included in the retrospective and prospective studies that we have already published.^1,12^ These studies received ethical approval from The Regional Ethics Committee IRCCS AOU San Martino – IST (CERLiguria) with Protocol number 503REG2015 (March 8, 2016) and 503REG2015 (September 12, 2018) respectively for retrospective and prospective study. All participating patients provided written informed consent, which included consent for publication of results. The preparation of the manuscript adhered to the STROBE guidelines, ensuring comprehensive and standardized reporting of our observational cross-sectional study (Supplemental File 2).^24^

In the retrospective study, CSF and serum of 262 neurological patients were collected between 2014 and 2015 from our laboratory.^1^ In the prospective study, CSF of 184 neurological patients from 6 Italian referral centers devoted to the management of immune-mediated neuropathies was collected starting from November 2018 and until the end of November 2019.^12^

In both studies all patients were subjected to spinal tap and blood withdrawal as part of the clinical practice within 1 week from their admission into the hospital, following the consensus recommendations of the Italian Association of Neuroimmunology for immunochemical CSF examination.^25^ One milliliter of CSF and serum were immediately stored at −80 °C for SM testing, to preserve lipid and protein integrity over time.^26^ We examined CSF and serum of patients authorizing access to personal health information according to the Helsinki Declaration as revised in 2013.

At entry, every patient underwent a neurological examination by an expert group of physicians. In addition, nerve conduction studies (NCS) were performed in the presence of suspected neuropathy. All the demographical, clinical, laboratory and neurophysiological data were previously reported in published papers.^1,12^ The patients included in this study were assigned to 3 major cohorts: (i) Active CIDP and Acute Inflammatory Demyelinating Polyneuropathy (AIDP; n = 61); (ii) other neurological disease (OND; n = 87), (iii) non-immune-mediated axonal neuropathies (n = 26). Active CIDP and AIDP include 18 AIDP, 34 Active typical CIDP, 9 active variants of CIDP. Definite CIDP, CIDP variants and AIDP were defined according to new published criteria.^27,28^ In particular, active CIDP was defined for those patients displaying a clinical relapse documented by the change of at least one point in the clinical scales at the moment of spinal tap. OND included hydrocephalus (n = 10), cerebrovascular disorders (n=13), cognitive impairment (n = 17), plexopathy or spondylosis (n = 7), cephalgia (n = 5), muscle disorders (n = 5), visual impairment (n = 5), cerebellar syndrome (n = 3), epilepsy (n = 3), cerebral tumor (n = 2), healthy patients (n = 14), motor neuron disease (n = 3). We also included in our study stable CIDP that was defined for those patients displaying unvaried clinical scores. Within this group (n = 18) there were 9 typical and 9 variants of CIDP. Suspected CIDP, not fulfilling new EAN/PNS guideline, were not included in the control group but analyzed as an independent group, also referred to as “NO EAN/PNS CIDP” (n = 31). This last group include neuropathy of unknown etiology (n = 11), axonal neuropathy with different comorbidities including diabetes (n = 8), hereditary neuropathy with pressure palsies (HNPP; n = 2), plexopathy (n = 3), multifocal motor neuropathy (n = 2), anti-myelin-associated glycoprotein neuropathy (n = 1), amyotrophic lateral sclerosis (n = 1), deficit of vitamin B12 (n = 1) and 2 patients with undefined diagnosis.

### Sphingomyelin Assay

The assay is based on lipid extraction from tissues, cells and biological fluids followed by enzymatic reactions consisting in the following steps: (1) hydrolysis of SM to phosphorylcholine and ceramide by sphingomyelinase; (2) hydrolysis of phosphorylcholine by alkaline phosphatase yielding choline and (3) oxidation of choline by choline oxidase with formation of hydrogen peroxide and betaine. Hydrogen peroxide, in the presence of horseradish peroxidase is able to react 1:1 with dihydroxyphenoxazine (AR) to generate resorufin, a highly fluorescent product.^11^

#### Lipid Extraction for SM Dosage

##### Detergent/Heating Method

Sciatic nerve, liver, spleen, lung were collected from WT and CMT1A rats, from CTRL and EAE mice and immediately frozen in liquid nitrogen and stored at −80°C. Tissues were weighted, homogenized in 0.25% Triton X-100 (20 mL/gr tissue) and centrifuged (10.000 × g for 5 minutes). Twenty μL of tissue homogenates were mixed to an equal volume of 0.25% Triton X-100 and then heated at 70°C for 5 minutes, cooled to RT, and briefly centrifuged in a microfuge. The supernatant was used for SM determination by a fluorescence-based assay (see below). For rat brain and mouse spinal cord, the protocol of extraction was the same, but these tissues were homogenized using 0.50% Triton X-100 instead of 0.25% to improve lipid recovery.

##### Bligh and Dyer Method

DRG homogenate (20 μL), human serum (10 μL), and CSF (450 μL) were mixed with CHCl_3_:CH_3_OH:H_2_O 1:2:1. Following centrifugation (5000 x *g* for 5 minutes), the lower organic phase was collected and dried out overnight at RT. Remains were dissolved in 20 to 30 μL of 0.25% Triton X-100 for SM determination.^29^

#### SM Dosage in 96-Well Microplates

All chemicals used in the following procedures were purchased by Sigma-Aldrich. In particular, 5 to 10μL of lipid extract from rat and tissue homogenates and DRG cultures, human CSF and serum were added to individual wells of a 96-well microtiter plate containing an enzymatic cocktail composed of 12.5 mU of *Bacillus cereus* sphingomyelinase (Sigma-Aldrich, S7651-10UN), 400 mU of alkaline phosphatase (Sigma-Aldrich, P6774-1KU), 120 mU of choline oxidase (Sigma-Aldrich, C5896-100UN), 200 mU of horseradish peroxidase (Sigma-Aldrich, P8125-5KU), and 20 nmol of AR (Sigma-Aldrich, 90101-5MG-F) in 100 μL of reaction buffer (50 mM Tris-HCl, 5 mM MgCl2, pH 7.4).

For each sample, the relative negative control obtained removing sphingomyelinase by the reaction mixture was also analyzed. After 20 minutes incubation at 37°C in the dark, the microtiter plate was read using a fluorescence microplate reader with excitation and emission wavelength at 560 and 587 nm, respectively (Infinite 200 PRO, Tecan Italia Srl). A standard curve was prepared by making serial dilutions (from 0.0125 to 1.6 nmol) from a 2.8 nmol/μL of SM standard stock solution. This curve showed both a linear regression from 0.2 to 1.6 nmol of input SM and high sensitivity at very low concentration (<0.02 nmol). At concentrations higher than 1.6 nmol the curve was less linear and fits a hyperbola regression equation underestimating the SM amount.

For each sample SM levels were calculated from the difference in fluorescence between the sample and its relative negative control. Resulting values were interpolated with the related standard curve to obtain absolute SM concentration (nmol).

### Quantification of Sphingomyelins by Lc-Ms/Ms

Targeted analysis of sphingomyelins in patients’ CSF for agreement analysis was performed following an already published protocol.^30^

### Statistical Analysis

Results are presented as mean ± SEM unless otherwise specified. Goodness of fit (*r*^2^) was determined by linear regression analysis. Statistical difference between 2 groups was determined using the two-tailed Student’s *t*-test. Multiple group comparison was performed by 1-way analysis of variance (ANOVA; followed by the Holm-Sidak test) and by 2-way ANOVA for SM content during development. Correlation coefficient was estimated by the Spearman’s rank correlation test. Agreement analysis between different methods was estimated by linear regression analysis. Statistical difference was considered to be significant when *P* < .05. All statistical analysis was performed using the Graph Pad V.9.0 (Prism) software.

## Results

### SM Monitors Myelin Deficit in a Dysmyelinating Neuropathy

In previous work we have already demonstrated that SM is specifically enriched in PNS, in particular sciatic nerves contain 5 times more SM than non-myelinated tissues (ie, liver, spleen and lung).^1^

**Figure 1.**
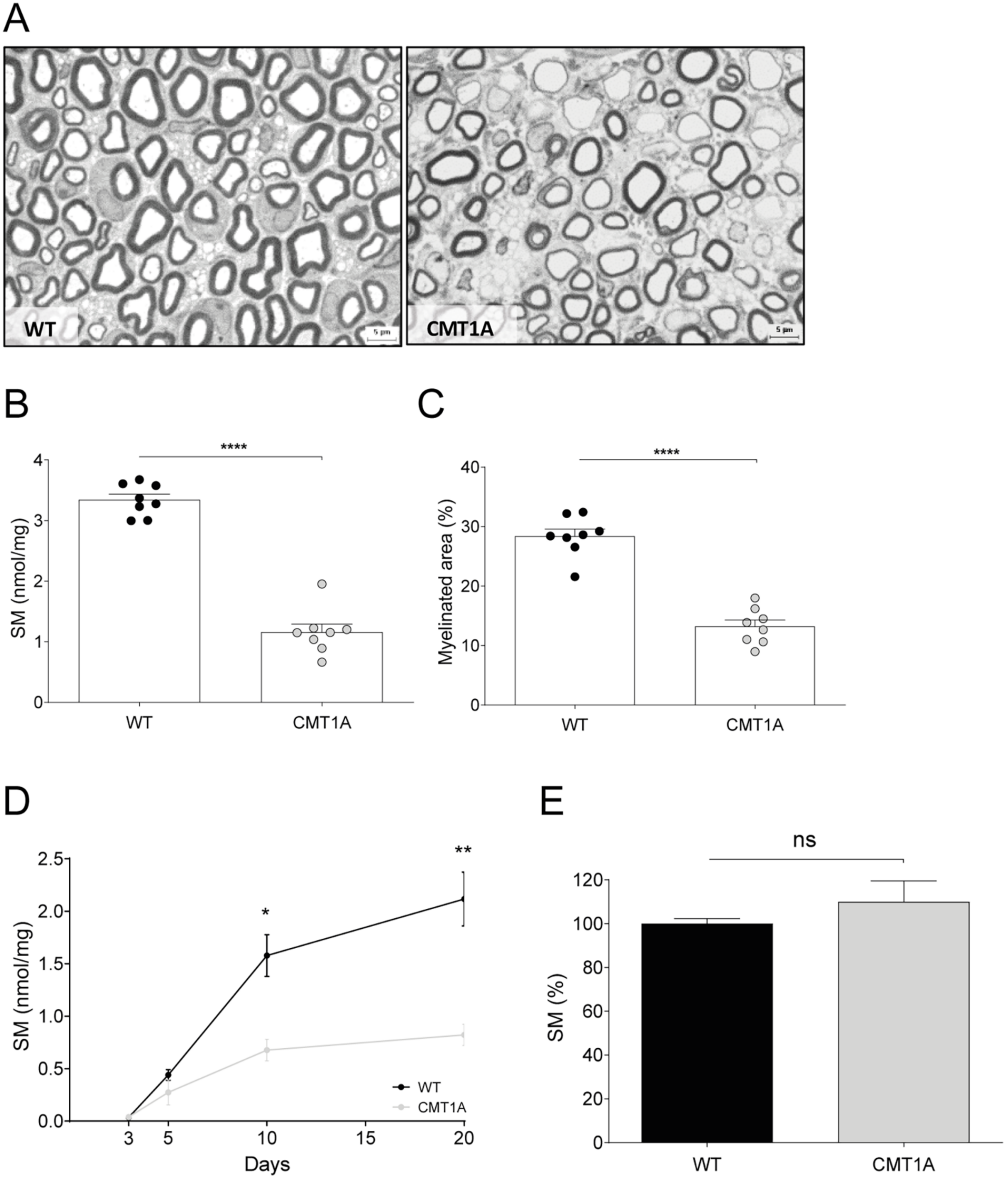
SM monitors myelin deficit in CMT1A: (A) representative images of blue toluidine cross section of sciatic nerves of 30-day-old WT and CMT1A rats, (B) we found a remarkable decrease of SM content in homogenate of sciatic nerves from 30-day-old CMT1A rats (n = 8) compared to controls (n = 8; WT vs CMT1A: 3.342 ± 0.093 vs 1.160 ± 0.1316 nmol/mg), (C) quantitative neuropathology performed on the contralateral sciatic nerves of the same animals displayed a significant reduction in the percentage of myelinated area (WT vs CMT1A: 28.40 ± 1.206 vs 13.24 ± 1.066%), (D) SM levels in rat sciatic nerves progressively increased during the critical timing of peripheral myelination and detects a very early shortage of this myelin lipid in CMT1A (WT vs CMT1A, n = 3: 3-day-old, 0.038 ± 0.0036 vs 0.0397 ± 0.0036 nmol/mg; 5-day-old, 0.44 ± 0.0503 vs 0.2744 ± 0.1197 nmol/mg; 10-day-old, 1.578 ± 0.1995 vs 0.6768 ± 0.1028 nmol/mg; 20-day-old, 2.116 ± 0.2566 vs 0.8222 ± 0.1013 nmol/mg), and (E) SM levels in brain of WT and CMT1A rats did not change (WT vs CMT1A, n = 3: 100.0 ± 2.358% vs 110.0 ± 9.525%). Statistical difference between 2 groups in B, C and E was determined using the two-tailed Student’s *t-*test. Multiple group comparison in D was performed by 2-way ANOVA. *P < .05, **P < .01, ****P < .0001.

To investigate whether SM dosage is able to detect peripheral myelinopathy, we quantified SM in a well-known dysmyelinating inherited disorder, the CMT1A neuropathy (Figure 1A).^14,31^ In particular, homogenate of sciatic nerves from 30-day-old CMT1A rats and wild-type (WT) littermates were used. We found a remarkable decrease of SM content in transgenic animals compared to controls (*P* < .0001; Figure 1B). Consistently, quantitative neuropathology performed on the contralateral sciatic nerves of the same animals displayed a significant reduction in the percentage of myelinated area (Figure 1C).

We also monitored SM levels in sciatic nerves during postnatal development at critical time points for rat peripheral myelin membrane biogenesis (ie, within 10-20 days after birth). We found that SM content monitors physiological maturation of PNS and detects a very early shortage of this myelin lipid in CMT1A (Figure 1D). Given that CMT1A is a congenital dysmyelinating disorder in which myelin is incorrectly formed and organized since the early stages of development, it is worth highlighting that SM dosage is sensitive and specific to identify and monitor this myelin deficit.^16^

Finally, given that CMT1A myelinopathy is almost restricted to PNS, we verified whether SM levels were consistent with this issue. Actually, SM dosage performed in normal and pathological condition highlighted a reduction of this lipid just in the sciatic nerves but not in the brain of the same rats (Figure 1E).

### SM is Detectable in Central Nervous System and Identifies Central Demyelination

To demonstrate that SM dosage is informative to track CNS myelin, we quantified this lipid in different myelinated tissues of adult mice.

**Figure 2.**
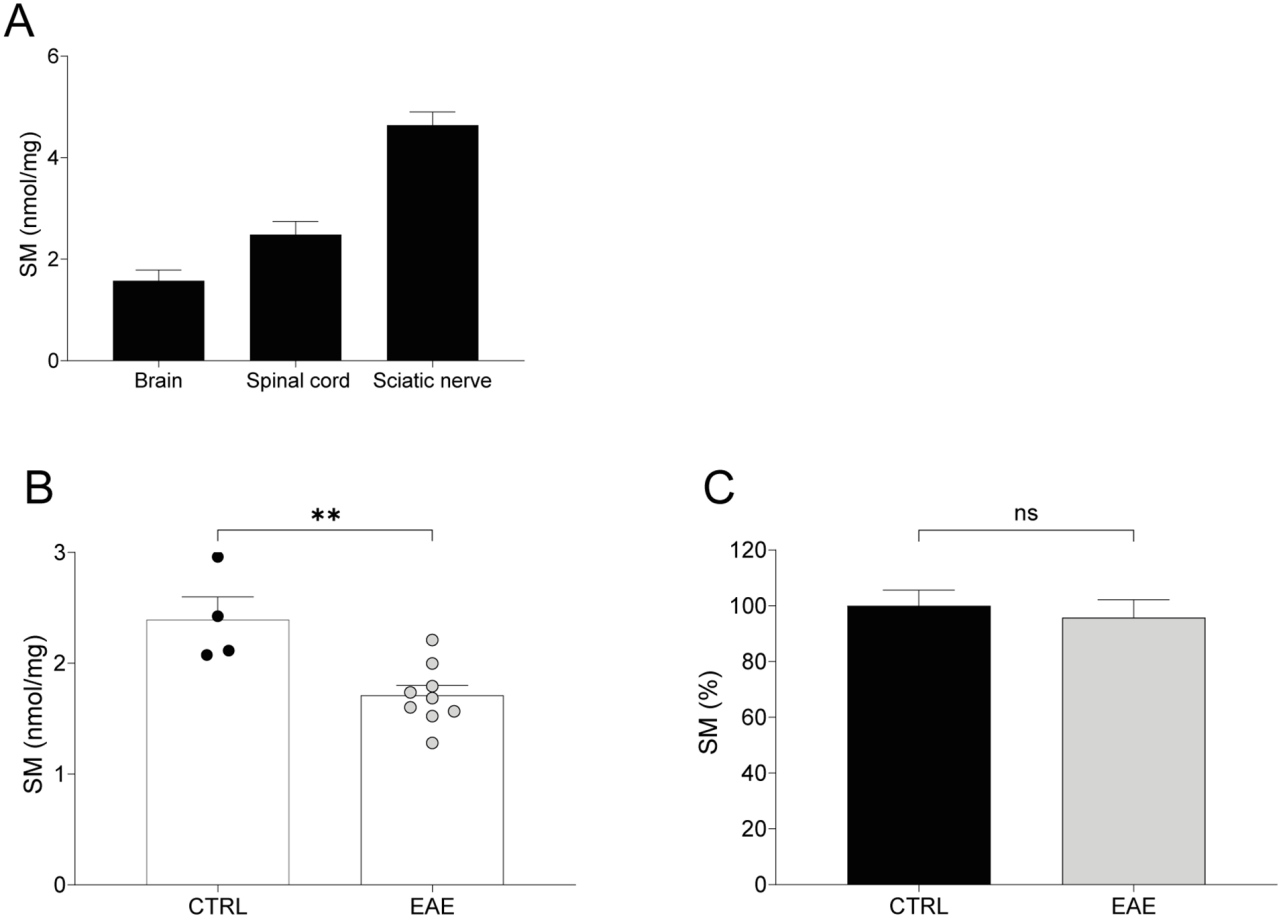
SM is detectable in central nervous system and identifies central demyelination: (A) SM is detectable both in brain and spinal cord of 210-day-old mice by our assay and at a lesser extent than in sciatic nerve (brain vs spinal cord vs sciatic nerve, n = 2: 1.579 ± 0.2085 vs 2.846 ± 0.2572 vs 4.641 ± 0.2590 nmol/mg), (B) In lumbar spinal cord of EAE mice (n = 9) we found a reduction of SM levels compared to CTRL (n = 4; CTRL vs EAE: 1.710 ± 0.090 vs 2.393 ± 0.2043 nmol/mg), and (C) Notably, in sciatic nerves of both EAE and control mice we did not find any difference in SM levels (CTRL vs EAE, n = 3: 100.0 ± 5.580% vs 95.84 ± 6.315%). Statistical difference between 2 groups in B and C was determined using the two-tailed Student’s *t*-test. **P < .01.

We found that SM is detectable both in brain and spinal cord by our assay and, as expected from the literature, at a lesser extent than in PNS (Figure 2A).^32^ To support the usefulness of SM assay in monitoring also central demyelination, we quantified it in the lumbar tract of spinal cord of mice in which experimental autoimmune encephalomyelitis (EAE) was induced.^20^ We found a significant decrease of SM compared with those of control littermates (*P* < .01; Figure 2B). Notably, in sciatic nerves of both EAE and control mice, where no signs of myelin pathology are present, we did not find any difference in SM levels (Figure 2C).

### SM Assay Monitors Small Myelin Rearrangements

**Figure 3.**
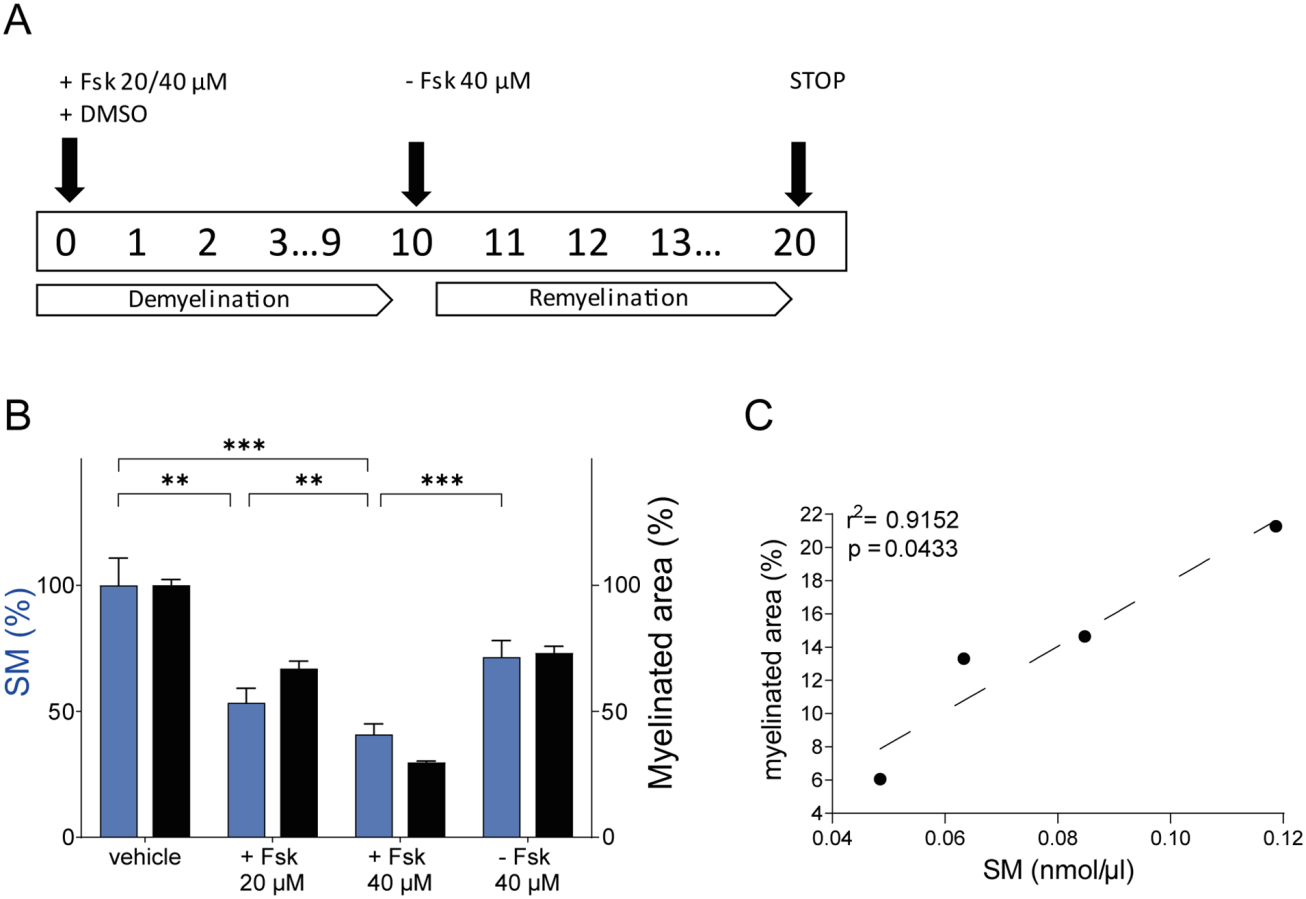
SM assay monitors small myelin rearrangements in an in vitro model of demyelination and remyelination: (A) schematic representation of the in vitro model of demyelination and remyelination that we used (see methods section), (B) SM dosage proved sensitive enough to detect both dose-dependent demyelination and remyelination (Vehicle vs +Fsk 20 μM vs +Fsk 40 μM vs −Fsk 40 μM, n = 6: 100 ± 2.309 vs 66.96 ± 2.982 vs 29.68 ± 0.5772 vs 73.11 ± 2.694%). This trend closely parallels myelin changes quantified as the percentage of myelinated area (ie, MBP-positive myelinated fibers) in each condition (Vehicle vs +Fsk 20 μM vs +Fsk 40 μM vs −Fsk 40 μM: 100 ± 2.309 vs 66.96 ± 2.982 vs 29.68 ± 0.5772 vs 73.11 ± 2.694%), (C) moreover, we found a statistically strong correlation between percentage of MBP positive area and SM amount (nmol/µl; *r*^2^ = .9152). Multiple group comparison was performed by 1-way analysis of variance (ANOVA) followed by the Holm-Sidak test in B. Correlation coefficient was estimated by the Spearman’s rank correlation test. **P < .01, ***P < .001.

After obtaining evidence that SM was especially informative for myelinated tissues of the PNS and CNS in physiological and pathological conditions, we further demonstrated its sensitivity and specificity as a myelin biomarker in an in vitro model of demyelination and remyelination.^21^ The protocol is detailed in the methods section and a timetable is summarized in Figure 3A. In particular, DRG cultures were treated with Fsk 20 and 40 μM to induce a progressive demyelination, and then let to recover by Fsk removal. SM dosage proved sensitive enough to detect both dose-dependent demyelination and remyelination (*P* < .0001; Figure 3B). This trend closely parallels myelin changes, namely the percentage of myelinated area (ie, MBP-positive myelinated fibers), in each condition (Figure 3B). Of note, we found a statistically strong correlation between the percentage of MBP positive area and SM amount (nmol/µl; *r*^2^ = .9152; Figure 3C).

### Optimization of SM Dosage in Human Biological Fluids

Given that SM dosage displayed a reliable monitoring of myelin in preclinical studies, we moved forward into the clinic, testing SM as myelin biomarker in biological fluids from neurological patients.

**Figure 4.**
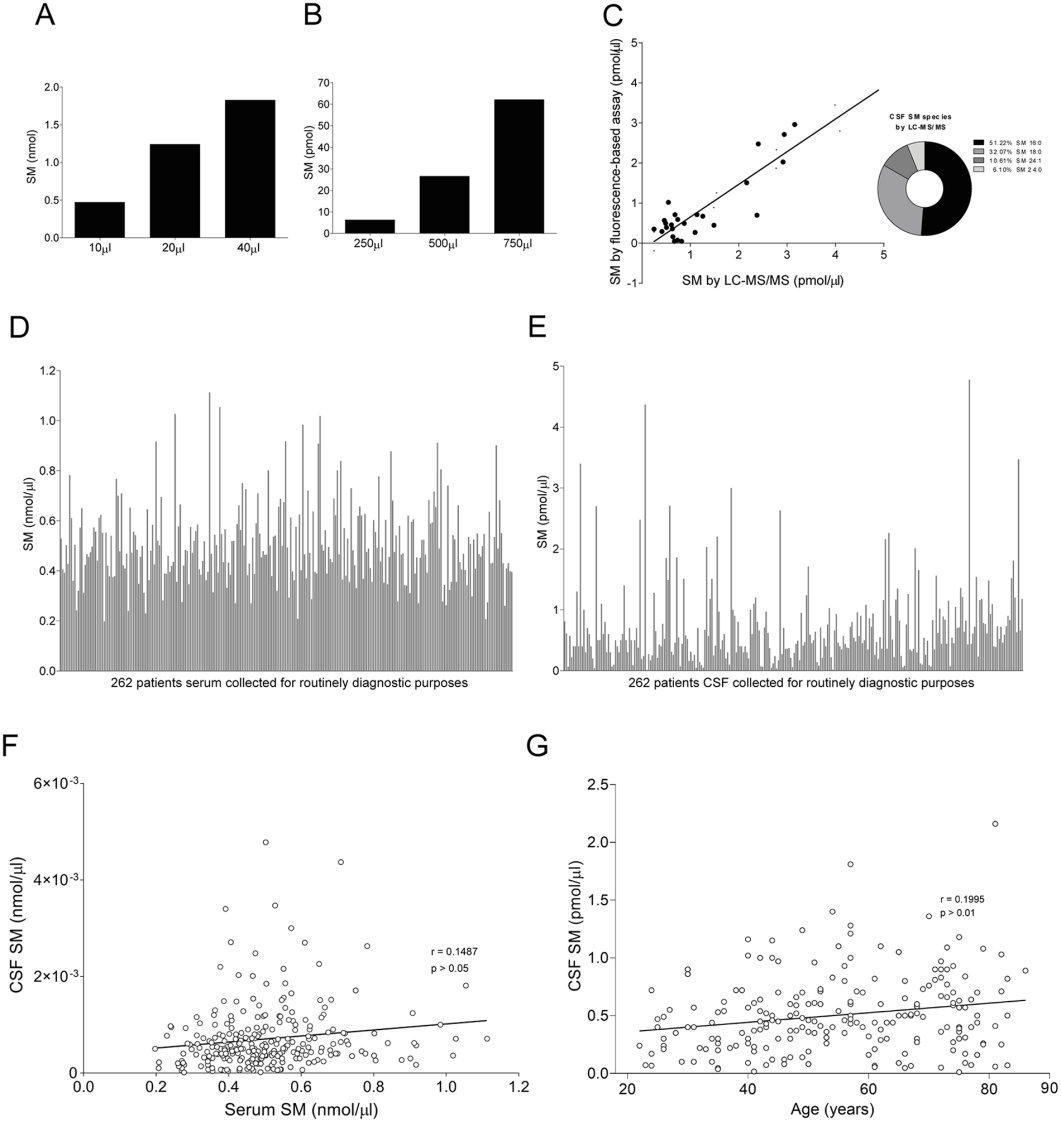
Optimization of SM dosage in human biological fluids: (A and B) we tested the sensitivity of the SM fluorescence-based assay, loading increasing volume of serum (A) and CSF (B) from a neurological patient. SM was detectable in both the biological fluids and increased proportionally to the volume of the sample, (C) to support the reliability of SM dosage, we performed an agreement analysis comparing CSF SM levels quantified by our fluorescence-based assay with those measured by LC-MS/MS in 31 neurological patients. Linear regression analysis displayed a linear relationship between the 2 methods, as outlined by a goodness of fit (*r*^2^) = 0.8448. SM values reported on x axis resulted from the sum of the major SM species detected by LC-MS/MS, namely SM 16:0 (51.22%), SM 18:0 (32.07%), SM 24:1 (10.61%) and SM 24:0 (6.1%), (D and E) SM levels quantified in the serum (D) and CSF (E) of 262 neurological patients were extremely variable to suggest a potential clinical relevance for patients affected by neurological disorders, (F) Correlation analysis between SM levels in the CSF and serum of 262 neurological patients did not show any correlation (*r* = .1487; *P* > .05), and (G) correlation analysis in the same cohort of patients between CSF SM levels and age did not show any correlation (*r* = .1995; *P* > .01). Linear relationship between the 2 methods in C was estimated by linear regression analysis. Correlation coefficient was estimated by the Spearman’s rank correlation test in F and G.

First, we verified the sensitivity of the SM fluorescence-based assay by loading increasing volumes of serum and cerebrospinal fluid (CSF) from a neurological patient (Figure 4A and B); SM was detectable in both biological fluids and increased proportionally to the input volume of the sample. Then, to assess the reliability of these results, we performed an agreement analysis comparing CSF SM levels quantified by the fluorescence-based assay with those measured by high-resolution mass spectrometry (ie, LC-MS/MS).^33^ Correlation results obtained in the CSF of 31 randomly selected neurological patients, displayed a linear relationship between the 2 methods (*r*^2^ = .8448; Figure 4C). SM values reported on x axis in Figure 4C resulted from the sum of the major SM species detected by LC-MS/MS, namely SM 16:0, SM 18:0, SM 24:1 and SM 24:0. The pie chart shows the contribution in percentage of each species to the total SM (Figure 4C).

Given that we found the fluorescence-based assay suitable to analyze SM levels in human biological fluids, we quantified SM in serum and CSF, collected for routine clinical practice, of 262 neurological patients. SM levels in these biological fluids displayed to be extremely heterogeneous among patients, especially in the CSF, thereby suggesting to be informative and deserving of further investigations (Figure 4D and E). We also found, in the same cohort of patients, that SM in the CSF did not correlate with that dosed in serum (Figure 4F); SM content in the serum is about 100 times higher than in the CSF and this result is important to exclude passive diffusion between the 2 compartments. Moreover, CSF SM did not correlate with the age of patients (Figure 4G).

### SM as a Biomarker of Myelin Breakdown in CSF of CIDP and AIDP

Based on the results obtained in experimental models and biological fluids from neurological patients, we hypothesized and demonstrated that demyelination at tissue level might be detected by SM dosage in the CSF of patients affected by immune-mediated demyelinating neuropathies, namely CIDP and AIDP.^34-37^

**Figure 5.**
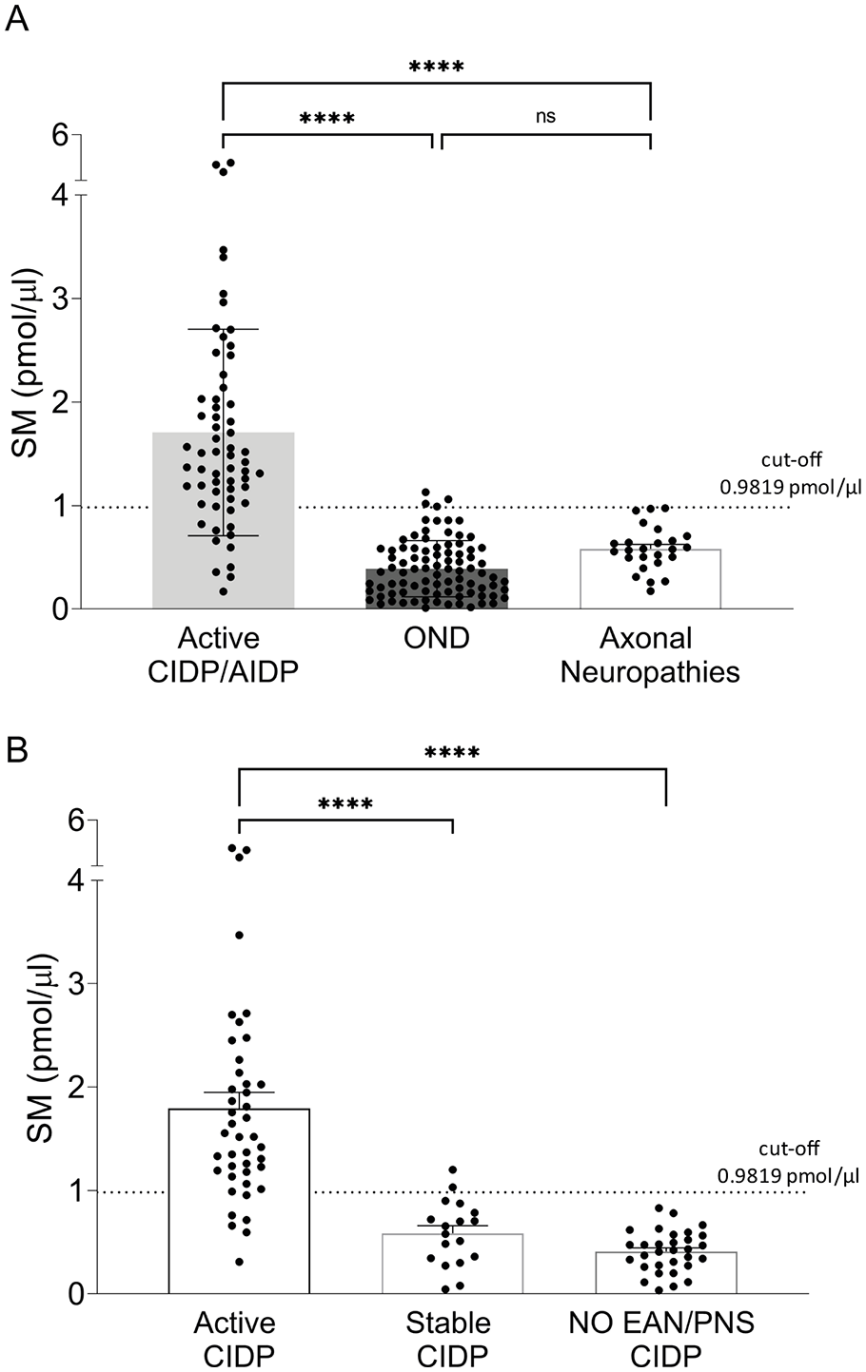
SM as a biomarker of demyelination in immune-mediated demyelinating neuropathies: (A) we assigned patients to 3 major cohorts, namely CIDP/AIDP (n = 61) OND (n = 87), and non-immune-mediated axonal neuropathies (n = 26). Using the validated cut-off for SM (0.9819 pmol/µL), we unambiguously identify demyelination typical of active CIDP and AIDP (Active CIDP/AIDP vs OND vs axonal neuropathies: 1.706 ± 0.1277 vs 0.3874 ± 0.029 vs 0.5811 ± 0.0041 pmol/µL), (B) SM levels differentiate active (n = 43) from stable (n = 18) stage of the disease and from suspected CIDP non-confirmed according to published criteria (NO EAN/PNS CIDP, n = 31; Active CIDP vs Stable CIDP vs NO EAN/PNS CIDP: 1.793 ± 0.1552 vs 0.5858 ± 0.0074 vs 0.4092 ± 0.1997 pmol/µL). Multiple group comparison was performed by 1-way analysis of variance (ANOVA) followed by the Holm-Sidak test. ****P < .0001.

We merged patient from monocentric retrospective and multicentre prospective studies and assigned them to 3 major cohorts, namely “active CIDP/AIDP” (n = 61), “other neurological diseases” (OND; n = 87), and “non-immune-mediated axonal neuropathies” (n = 26).^27,28^ Then, we used the validated cutoff for SM (0.9819 pmol/µL) to compare these groups, displaying the strength of our assay to unambiguously identify demyelination typical of active CIDP and AIDP (*P* < .0001; Figure 5A). Moreover, SM levels differentiate active from stable stage of the disease (*P* < .0001), fundamental to select the most appropriate timing for therapeutic intervention (Figure 5B).

Even more interesting, SM dosage allows to avoid misdiagnosis with a 100% specificity (*P* < .0001; Figure 5B).

## Discussion

This study displays the activities we engaged to fine-tune SM fluorescence-based assay as a novel myelin biomarker. From the research point of view, wieldy approaches are required to detect myelin changes in animal and in vitro models used to investigate the molecular mechanisms underlying myelination, demyelination and remyelination, and to test safety and effectiveness of pro-myelinating therapeutic options.^38-40^ From the clinical point of view, biomarkers that accurately reflect myelin disease pathology are extremely important for diagnosis, staging and monitoring treatment response, either in case in which therapies are already in use and in diseases in which pharmacological and non-pharmacological approaches are becoming available^9,41^

On this premise and given that identification and real-world application of novel fluid biomarkers into clinical practice require several aspects to be systematically addressed, including exploratory and technical studies using appropriate experimental settings, clinical retrospective studies and prospective validation studies, we assumed this roadmap to develop a novel myelin biomarker.^10^

The first task was to demonstrate that SM dosage identifies myelinated tissues of PNS and CNS both in physiological and pathological conditions. Actually, SM is specifically enriched in sciatic nerve, brain and spinal cord of WT rat and mouse (Figure 2A) and monitors myelination during postnatal development (Figure 1D).^1^ To further strengthen the issue, we found that our assay detects myelin deficit in a congenital dysmyelinating disorder, namely CMT1A, in which myelin is improperly formed and organized since the early stages of development (Figure 1B and D).^16^ Moreover, in a mouse model of CNS demyelination, namely EAE, SM dosage in spinal cord promptly recognizes this pathological hint (Figure 2B).

Of note, the high sensitivity of this assay is also supported by the results we obtained in a chemically induced in vitro model of demyelination/remyelination, where very small myelin rearrangements were detected (Figure 3A and B). This peculiarity makes SM assay an excellent tool for monitoring and quantifying myelin changes in in vitro systems used to screen potential promyelinating compounds.

Considering our results on experimental models essential exploratory and discovery activities to validate SM dosage as a myelin biomarker, we moved to biological fluids of neurological patients.

Indeed, body fluid biomarkers display most of the makings required to improve the management of neurological patients in a clinical setting, including the ability to support diagnosis, staging and progression of the disease, and with the evaluation of treatment efficacy. In particular, CSF is a primary matrix of interest in biomarker discovery, due to its direct contact with the nervous system and because it has been already shown to be useful in the diagnosis of neurological diseases including multiple sclerosis, Alzheimer disease, and infectious diseases of the CNS.^9,10^ In spite of that, not many novel promising biomarkers were identified, validated and implemented for clinical purposes in the last years, due to several gaps in their development and implementation processes.^10,41,42^

Therefore, to validate our hypothesis of SM in a clinical setting, we analyzed CSF from patients affected by CIDP and AIDP; in fact, proximal nerve roots located in the subarachnoid region are floating freely in the CSF and are in close contact with CSF. Therefore, the altered composition of CSF in these patients could mirror the myelin damage within the tissue of the nervous system.^34,43,44^ Even more, for these neuropathies, besides clinical and neurophysiological criteria, there are not clinically acceptable biological markers and misdiagnosis is still a major concern implying medical, social and economic consequences.^45-52^

In particular, we first optimized our fluorescence-based assay in the CSF and serum of these patients showing that SM is detectable in both these biological fluids (Figure 5A and B). Our results also displayed that SM levels are extremely variable among neurological patients and thereby potentially informative, especially in the CSF (Figure 5). Moreover, although SM levels in the serum are much higher than those detected in the CSF of the same patient, there was no correlation between the 2 values to underline the lack of influence between the 2 compartments fundamental to exclude a passive diffusion of SM from the periphery (Figure 4F).

Of note, the agreement analysis of CSF SM levels quantified by the fluorescence-based assay with those measured by high-resolution mass spectrometry displayed a linear relationship between the 2 methods (Figure 4C). Indeed, LC-MS/MS is an advanced technique with high sensitivity to detect and quantify any metabolite but is poorly acceptable in the routine clinical practice due to the high costs and specific skills required. To this end, high-resolution biomarker technologies, including mass spectrometry, have accelerated the discovery of novel biomarker candidates but few of them progressed up to clinical validation and development into clinical tests.^42^ Instead, SM assay that we have demonstrated to be in line with much more expensive techniques (ie, LC-MS/MS), is economically convenient, with a total estimated cost of 5 euro/sample; moreover, being easily amenable to routine utilization without the need for sophisticated equipment or operator skill, it is ready for widespread acceptance.

After demonstrating reliability, sensitivity and practicality of the SM assay in human biological fluids, we moved from the analytical to clinical validation. According to the recommendations of CSF society, a mandatory requirement before clinical implementation is a strong validation in wide and independent cohorts of patients.^9,10,26^ To this end, here SM data from neurological patients from different neurological centers, enrolled for retrospective and prospective studies, were merged and reanalyzed. In our previous studies we have already demonstrated that SM dosage is independent of CSF indexes used in the daily clinical practice and that SM cut-off for optimum sensitivity and specificity was 0.9819 pmol/µL.^1,12^

When we assigned patients to 3 major cohorts, namely “active CIDP/AIDP,” “OND” and “non-immune-mediated axonal neuropathies,” using the validated cut-off, our assay unambiguously identified demyelination (Figure 5A). SM dosage also displayed the ability to overcome other difficulties in the management of patients affected by CIDP.^49,51,52^ Indeed, SM levels differentiate active from stable stage of the disease fundamental to select the most appropriate timing for therapeutic intervention thereby improving pharmacovigilance of CIDP patients (Figure 5B). Moreover, CIDP patients in the stable stage of the disease become non-responder to therapy and, in that sense, SM dosage may allow to identify patients who do not require further treatment, avoiding severe side effects and associated costs of a prolonged unnecessary therapy.^53,54^ Among “Active CIDP” patients, five patients displayed SM levels under the cut-off; interestingly, these patients had a moderate clinical impairment mainly characterized by a distal sensory involvement at the neurophysiological examination. Of course, to confirm this aspect a very large cohort of patients is necessary. Lastly, SM dosage avoids misdiagnosis with 100% specificity being much more specific than the currently used laboratory gold standard test represented by CSF protein and albumin dosage.^12,55,56^

## Conclusion

SM assay encompasses most of the ideal characteristics of a biomarker representing a promising tool to the real-word monitoring of myelin changes in a wide spectrum of conditions. We are confident that this myelin biomarker might definitely improve the management of patients affected by myelin diseases in any medical assistance context.

## Supplemental Material

sj-pdf-1-bmi-10.1177_11772719251349605 – Supplemental material for A Comprehensive Description of the Roadmap to Identify and Validate a Myelin Biomarker Supplemental material, sj-pdf-1-bmi-10.1177_11772719251349605 for A Comprehensive Description of the Roadmap to Identify and Validate a Myelin Biomarker by Giovanna Capodivento, Davide Visigalli, Andrea Armirotti, Chiara Demichelis, Marinella Carpo, Roberto Fancellu, Erika Schirinzi, Daniele Severi, Diego Franciotta, Fiore Manganelli, Gabriele Siciliano, Alessandro Beronio, Elisabetta Capello, Paola Lanteri, Eduardo Nobile-Orazio, Angelo Schenone, Luana Benedetti and Lucilla Nobbio in Biomarker Insights

sj-pdf-2-bmi-10.1177_11772719251349605 – Supplemental material for A Comprehensive Description of the Roadmap to Identify and Validate a Myelin Biomarker Supplemental material, sj-pdf-2-bmi-10.1177_11772719251349605 for A Comprehensive Description of the Roadmap to Identify and Validate a Myelin Biomarker by Giovanna Capodivento, Davide Visigalli, Andrea Armirotti, Chiara Demichelis, Marinella Carpo, Roberto Fancellu, Erika Schirinzi, Daniele Severi, Diego Franciotta, Fiore Manganelli, Gabriele Siciliano, Alessandro Beronio, Elisabetta Capello, Paola Lanteri, Eduardo Nobile-Orazio, Angelo Schenone, Luana Benedetti and Lucilla Nobbio in Biomarker Insights
